# Sulfonimide-Based Dendrimers: Progress in Synthesis, Characterization, and Potential Applications

**DOI:** 10.3390/polym12122987

**Published:** 2020-12-15

**Authors:** Julia V. Bondareva, Stanislav A. Evlashin, Oleg V. Lukin

**Affiliations:** 1 Skolkovo Institute of Science and Technology, Bolshoy Boulevard 30, bld. 1, 121205 Moscow, Russia; s.evlashin@skoltech.ru; 2 Life Chemicals Inc., 5 Murmanskaya St., 02660 Kiev, Ukraine; olukin509@gmail.com

**Keywords:** dendrimers, sulfonimide, divergent approach, functionalization, generations, dendrons, dendritic polymers

## Abstract

There are more than 50 families of dendrimers, and some of which, such as polyamidoamine PAMAM, are well studied, and some are just starting to attract the attention of researchers. One promising type of dendrimers is sulfonimide-based dendrimers (SBDs). To date, SBDs are used in organic synthesis as starting reagents for the convergent synthesis of higher generations dendrimers, in materials science as alternative electrolyte solutions for fuel cells, and in medicinal chemistry as potential substances for drug transfer procedures. Despite the fact that most dendrimers are amorphous substances among the SBDs, several structures are distinguished that are prone to the formation of crystalline solids with melting points in the range of 120–250 °C. Similar to those of other dendrimers, the chemical and physical properties of SBDs depend on their outer shell, which is formed by functional groups. To date, SBDs decorated with end groups such as naphthyl, nitro, methyl, and methoxy have been successfully synthesized, and each of these groups gives the dendrimers specific properties. Analysis of the structure of SBD, their synthesis methods, and applications currently available in the literature reveals that these dendrimers have not yet been fully explored.

## 1. Introduction

**Figure 1:**
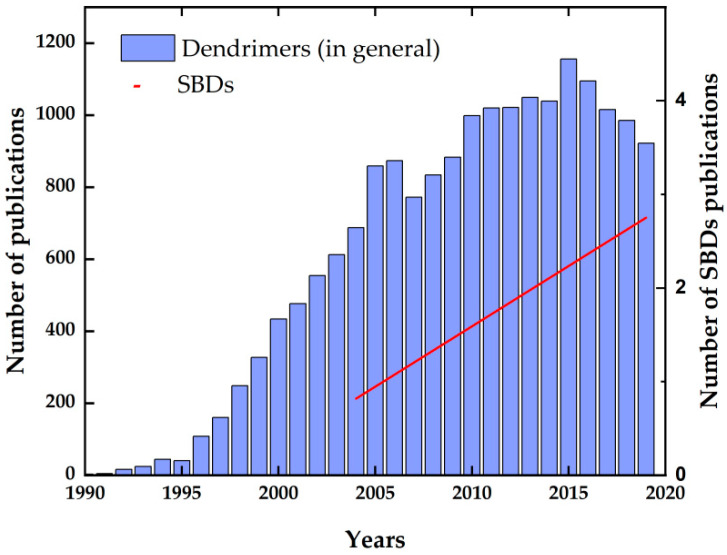
The number of publications related to dendrimers and sulfonimide-based dendrimers (SBDs) according to Scopus 1990–2020.

One of the long-term goals of basic science is the search for as many new and outstanding substances as possible under the limits of natural laws. Of course, new chemical substances are not synthesized by random approaches with no purpose in mind. Chemists create new materials with the aim that their properties will be scientifically significant or useful for practical purposes. The synthesis of new molecules is achieved by performing chemical reactions, some of which are already well known and some of which must be developed. Thus, another goal of basic science is to develop new synthetic strategies to create exciting and useful substances. One such recently prepared class of substances is dendrimers. Dendrimers have been studied for the past thirty years. The number of publications related to dendrimer research is growing every year, as shown in Figure 1, and this trend is expected to continue. 

**Figure 2:**
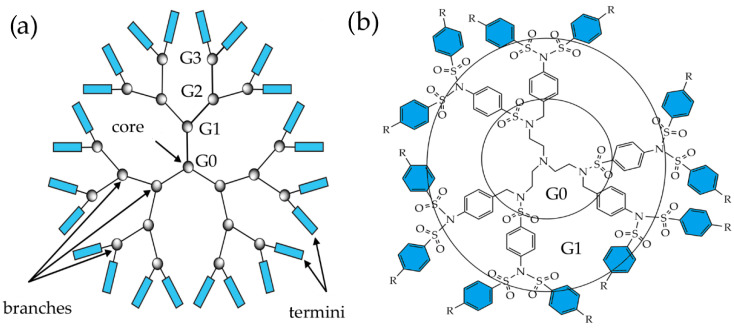
(**a**) Schematic of the dendrimer structure showing the key components. (**b**) A schematic of a G1 of SBD in the form of a planar graph.

This growing interest can happen because dendrimers are important in chemistry, biology, medicine, biotechnology, and the pharmaceutical and food industries [1]. Their physicochemical properties, such as monodispersity, multivalency, and globular shape, distinguish them from classic polymers and make them a separate class of molecules. Due to their inner cavities, dendrimers can create a suitable environment for the stabilization of nanoparticles, which can be directly synthesized from metal salts [2]. Despite these properties, there are a few commercial products based on dendrimers or using dendrimers as part of chemical composition [3]; some of them are already on the market, and some are in clinical trials [4,5]. The main reason for this is the dearth of synthetic strategies for preparing dendrimers. The absence of a highly efficient synthetic method and the difficulties associated with purifying increasingly branched macromolecules have inhibited their transfer from academia to industry. Figure 2a [6] shows a two-dimensional model of the dendritic structure in a flat form, consisting of a set of geometric figures representing the kernel, points of branches, and end groups [7]. Thus, many dendrimers have been constructed incorporating different functionalities either at the core, branches, terminals, or a mixture of any two or three of these dendritic units. Currently, there are about 50 dendrimer families, including PAMAM [8], poly(amidoamine-organosilicon) (PAMAMOS) [9], poly(propyleneimine) (PPI) [10], liquid crystal dendrimers [11], chiral dendrimers [12], peptide dendrimers [13], tecto-dendrimer [14], glycodendrimers [15], polyethylene glycol (PEG) dendrimers [16], etc. Since many dendrimers have been already obtained and investigated, the amount of information available in the literature is continuously growing. Therefore, we mainly focus on a specific type of dendrimer molecule, namely, sulfonimide-based dendrimers (SBDs), which contain sulfonimide groups at each branching point. The typical structure of an SBD is presented in Figure 2b, where a nitrogen atom represents the core and the branching points contain sulfonimide functionalities. 

Each structural part of the dendrimer is responsible for certain properties. The multiplicity of the core and the branching units determine the shape and size of the dendrimer. In some cases, the core can assign a particular function to the dendrimer. Increasing the number of branches increases the number of generations, which refers to the number of layers attached to the core. Moreover, the nature of the branching points influences the stability of the molecule itself and the presence of internal voids, which can accommodate guest molecules. The end groups create the outer shell of the molecule and give the dendrimer unique properties, such as low/high solubility in different solvents, viscosity, and conformational stability [17]. One of the advantages of dendrimers, along with other classes of macromolecules, is the presence of an enormous number of terminal groups, which exponentially amplify the desired properties. SBDs were chosen as the materials of interest due to their unique properties, which are absent in other similar branched macromolecules. Among polybranched compounds, SBDs possess relatively high melting (120–250 °C) and glass transition temperatures (135–162 °C) [7]. Additionally, aromatic sulfonimides are chemically stable and crystalline. The combination of these properties makes them applicable in situations where the material must be stable in the temperatures range of 120–130 °C. The main advantage of dendrimers is the ability to access the desired features through careful design and installing various chemical groups in the dendrimer. Furthermore, SBDs can be considered in terms of surface chemistry as functional surface coatings. [18] This review primarily focuses on the advances related to the synthesis, characterization, and applications of SBDs in the last few years. The aim of review is to draw attention to this promising type of dendrimer and highlight some obstacles that still need to be overcome in this field.

## 2. Properties

### 2.1. Structure

In terms of size, dendrimers are organic nanoparticles (as liposomes, carbon nanomaterials, and polymer micelles are) [19]. As nanoparticles, their size does not exceed 15 nm, and they show unique size-dependent physical and chemical properties. For example, the dimensions of recently synthesized SBDs up to the G5 do not exceed 5 nm, as shown in Table 1 [20].

The properties of sulfonimide groups are not well understood, and they are described in the literature relative to a tautomer of a sulfonamide group [21,22]. 

In organic chemistry, a sulfonimide is a functional group consisting of two sulfonyl groups (–SO_2_) bound to a nitrogen atom from one side and carbon from another side. The compounds are structurally similar to acid anhydrides. The basic nitrogen atom of a sulfonimide with a lone pair can participate in hydrogen bonding [23]. The sulfonyl group is a relatively inactive functional group characterized as weak basic, where a sulfur atom is doubly bonded to two oxygen atoms [24]. The reaction of a sulfonyl chloride with ammonia or an amine leads first to a sulfonamide, and in the case of full nucleophilic substitution, a sulfonimide can be obtained. Sulfone (–SO_2_) and sulfonamide (–SO_2_NH) groups can be found in drug-like molecules and are used in pharmaceuticals due to their antibacterial, antimicrobial, antidiabetic, and antiretroviral activities [25]. 

### 2.2. Isomeric Structures

The structure, number of branches, and molar weight directly affect the properties of SBDs. According to the topology, dendrimers can be considered as isographic dendrimers, having the same branches (symmetrical structure), or non-isographic dendritic isomers, containing different branches (asymmetric structure). There is some structure–property correlation. To this extent, several sulfonimide-based isomers with different molecular weights and shapes have been evaluated. Several properties such as melting temperature, solubility, chromatographic separation, nuclear magnetic resonance (NMR), spectroscopic, and mass spectrometric characteristics of these compounds were analyzed [7]. The low-molar-weight dendrimers with mostly linear shapes and four to six peripheral groups have high melting points and low solubility in chloroform (CHCl_3_) [7]. The melting points and solubility of high-molar mass dendrimers with seven, eight, and ten peripheral groups differ noticeably less. Thus, with an increase in dendrimer generation, fewer differences in the properties listed above are observed between dendrimer isomers. The formation of a dense spherical packing of high-generation dendrimers, in which the outer shell mainly determines the properties, is one possible explanation for this behavior. 

### 2.3. Functional Groups

Aromatic SBDs structures contain peripheral methyl and nitro groups that can be chemically converted to carboxylic acids and amines for further chemical or biological modification. From the point of view of medicine, functional groups provide additional binding sites, specific properties, and behaviors responsible for biological activities. For a given drug molecule, its functional groups affect its general solubility in aqueous solutions, the ability to specifically bind to biological targets, mechanism and duration of action, etc. [26,27]. Carboxylic and amino functional groups may provide an additional opportunity for the formation of a covalent bond with a biological target, enzyme, or drug molecule, or they may participate in the formation of hydrogen bonds [28,29,30,31]. 

Adding even one chemical group to a given molecule can change some dendrimer properties, such as the solubility, electronegativity, and conformation of the molecule. When a dendrimer structure is functionalized with photoactive units [32,33], it can exhibit moderate fluorescent activity and be used as a photoactive tracer. The properties of a dendrimer that contains photoactive chemical parts depend on the interactions with the ground and excited states. For example, it has recently been shown that dendrimers with a cyclam core and naphthyl-based branches exhibit three types of emission bands [34]. Additionally, poly(propylene amine) dendrimers modified on the periphery with fluorescent naphthyl sulfonamide groups can be involved in the coordination of metal ions [32].

**Figure 3:**
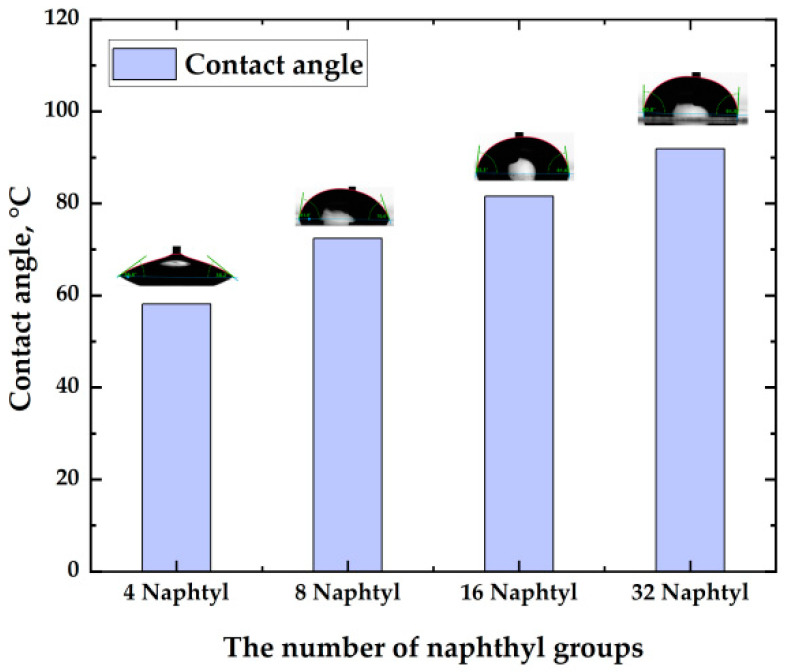
The dependence of contact angle on the number of naphthyl terminal groups in the dendrimer structure.

The presence of large alkyl chains or aromatic systems such as naphthalene makes the dendrimer lipid-soluble. Changing the number of such groups makes it possible to control the hydrophobicity of the dendrimers. Thus, the outer shell of SBDs can be functionalized with naphthalene terminal groups (from four to 32 groups for the G5 dendrimer, which has been synthesized for the first time) [20]. As naphthyl groups, along with long alkyl chains, make the molecule hydrophobic, it is possible to create a water-repellent film [18]. Additionally, the presence of photoactive naphthyl groups, which can form intramolecular or intermolecular bonds under UV radiation, allows creating films with different types of binding and packaging. So, under UV irradiation, naphthyl groups can be dimerized with the formation of additional covalent bonds, in contrast to unirradiated UV samples, where the non-covalent bond predominates. Thus, the number of naphthyl groups, the use of UV radiation, and the formation of additional bonds create a dense packing of molecules and lead to different contact angle values, as shown in Figure 3.

### 2.4. Molar Masses

The focal point of the dendritic polymer can impart some interesting properties. For example, if dendrons carry a polymerizable unit, this could allow solid-state polymerizations [35]. The obtained sulfonimide dendrons, which contain a methacrylate group in the focal point, can be polymerized [36] and reached the molar masses of the G1 and G2 polymers *M*_w_ = 1500 and *M*_w_ = 1600 kDa, respectively. All such polymers are soluble in polar solvents, such as dimethylsulfoxide (DMSO) and dimethylformamide (DMF) and are insoluble in hexane. 

### 2.5. Solubility

Dendrimers have multifunctional surfaces that allow their solubility and interactions with other molecules to be tuned for different environments. Solubility is crucial for biomedical applications. To be applied in the pharmaceutical field, dendrimers should be soluble in biologically safe solvents. The solvent must be entirely safe for humans and effectively dissolve the dendrimers. One such biologically compatible solvent is water. Water is the most advantageous solvent in many respects, e.g., its availability, biocompatibility, and low price, and it does not require additional stages of removal and cleaning. SBDs are usually insoluble in water. Some dendrimers can be solubilized if a solubilizing agent is added. SBDs are soluble in CHCl_3_, but this solvent is toxic. Another suitable solvent for SBDs is DMSO. DMSO is considered to have a low toxicity that can dissolve both polar and nonpolar compounds. At the same time, DMSO can easily penetrate the skin along with the solute, which may cause undesirable effects. In some cases, binary mixtures of solvents are used, e.g., a popular mixture is DMSO and water. The main limitation with SBDs in this regard is that they are not soluble in water, which makes it difficult to use such materials in biomedical fields. In this context, the development of a method for the aqueous solubilization of these dendrimers by adding solubilizing agents such as cyclodextrins [37] is a target for future studies.

SBDs and dendrons of all generations bearing peripheral tosyl- and 2-naphthyl sulfonyl groups exhibit very high solubility in both polar aprotic and nonpolar aprotic solvents (except for hexane). Their solubility in DMF, tetrahydrofuran (THF), CHCl_3_, and benzene are in the range of 150–250 mg/mL [6]. Nosyl-decorated branched sulfonimides have poor solubility in the above-mentioned solvents at room temperature except for G1 species and Janus-type dendrimers. Compounds with peripheral nitro groups can be dissolved in hot THF and hot CHCl_3_. All the compounds, as mentioned above, have low or no solubility in methanol (MeOH) and ethanol (EtOH). Oligosulfonimides bearing peripheral amino groups are soluble in alcohols or mixtures of alcohols with either chlorinated solvents or benzene. 

### 2.6. Voids and Cavities

**Figure 4:**
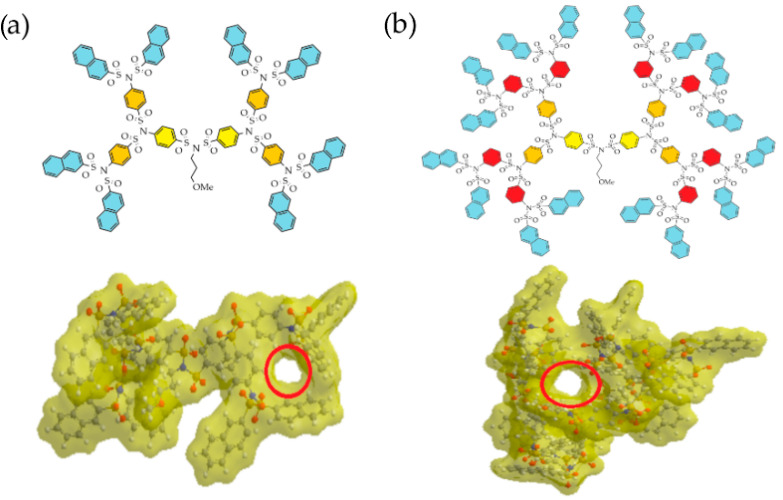
(**a**) Planar representation of a G3 SBD and its 3D architecture with a Connolly molecular surface. (**b**) Planar representation of a G4 SBD and its 3D architecture with a Connolly molecular surface.

Additionally, the presence of internal voids is an important parameter of dendrimers. Internal cavities can serve as reservoirs for the storage of small molecules and nanoparticles, allowing dendrimers to participate in host–guest chemistry. Figure 4 shows the example of G3 and G4 naphthyl decorated SBDs, and their internal cavities are marked with red circles. 

Dendrimers can interact with low molecular weight drug molecules in several ways: physical encapsulation, covalent binding through terminal chemical groups, and electrostatic interaction [38]. The possibility of using all these binding methods are explained by the dendrimer’s structural features, such as the presence of internal cavities for placing the drug inside the structure of the dendrimer and the existence of many surface functional groups for covalent conjugation with the drug substance. 

## 3. Synthesis

Branched cascade molecules called dendrimers were first synthesized in 1978 [39]. Since then, dendrimer molecules have received extensive attention in organic, supramolecular, and polymer chemistry. For SBDs, two synthetic approaches are often used, which are known as divergent and convergent strategies, but several new techniques have also been applied in recent years. 

### 3.1. Divergent Technique

The first attempt at the synthesis of SBDs was reported by Prof. Vögtle et al., who obtained sulfonamide-substituted G2-G4 polypropylene amine (POPAM) dendrimers by N-monosulfonylation and N-bissulfonylation [40]. Prof. Vögtle et al. proposed the use of sulfonic acid chlorides (sulfonyl chlorides) for the mono- and bifunctionalization of dendrimers. Arylsulfonyl chlorides are commercially available, making these dendrimers synthetically accessible and increasing the potential structural variability of dendritic sulfonimides. They reported the first N-sulfonylation of poly(propyleneimine) (PPI) dendrimers and suggested using this method to synthesize higher-generation SBDs [32].

**Scheme 1:**
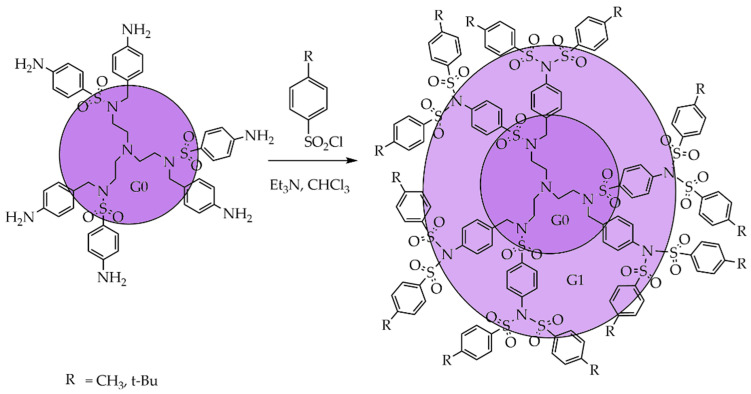
Controllable per-functionalization (persulfonylation) of dendritic oligoamines.

The next synthetic approach for obtaining dendrimers with sulfonimide units at the branching points was reported by Prof. Vögtle et al. in 2004 [41]. Prof. Vögtle et al. developed a method that allows the controlled functionalization of oligoamines such as tris(2-aminoethyl) amine by persulfonylation using sulfonyl chlorides. Thus, the sulfonylation reaction can be controlled by changing the reaction conditions, for example, the amount and nature of the reagents as well as the time and temperature of the reaction. The selectivity of the persulfonylation has opened up possibilities for the synthesis of new types of SBDs with more complex structures and different functional groups through sulfonylation of the amine, by not only the addition method 1→1, but also using the more effective way 1→2 [42]. The selectivity of the sulfonylation reaction may also depend on the nature of the base used, for example, the reaction of tris (2-aminoethyl) amine with arylsulfonyl chlorides in dichloromethane (CH_2_Cl_2_) or CHCl_3_ in the presence of a base catalyst leads to the formation of sulfonamides according to the addition scheme 1→1. The same reaction can lead to sulfonimide according to the 1→2 addition scheme if cesium carbonate is used as the base. The yields of sulfonimide depend on the nature of the sulfonyl chloride. Along with the influence of the base’s nature, the choice of sulfonyl chloride affects the sulfonylation reaction. Scheme 1 shows that the sulfonylation of a sulfonamide with six terminal amino groups using sulfonyl chlorides containing methyl and tert-butyl groups in the presence of triethylamine unexpectedly allowed the sulfonylation of all external amino groups. Thus, the method using the 1→2 branching scheme will enable one to quickly and efficiently obtain chemically stable dendrimers with sulfonimide branches.

**Scheme 2:**
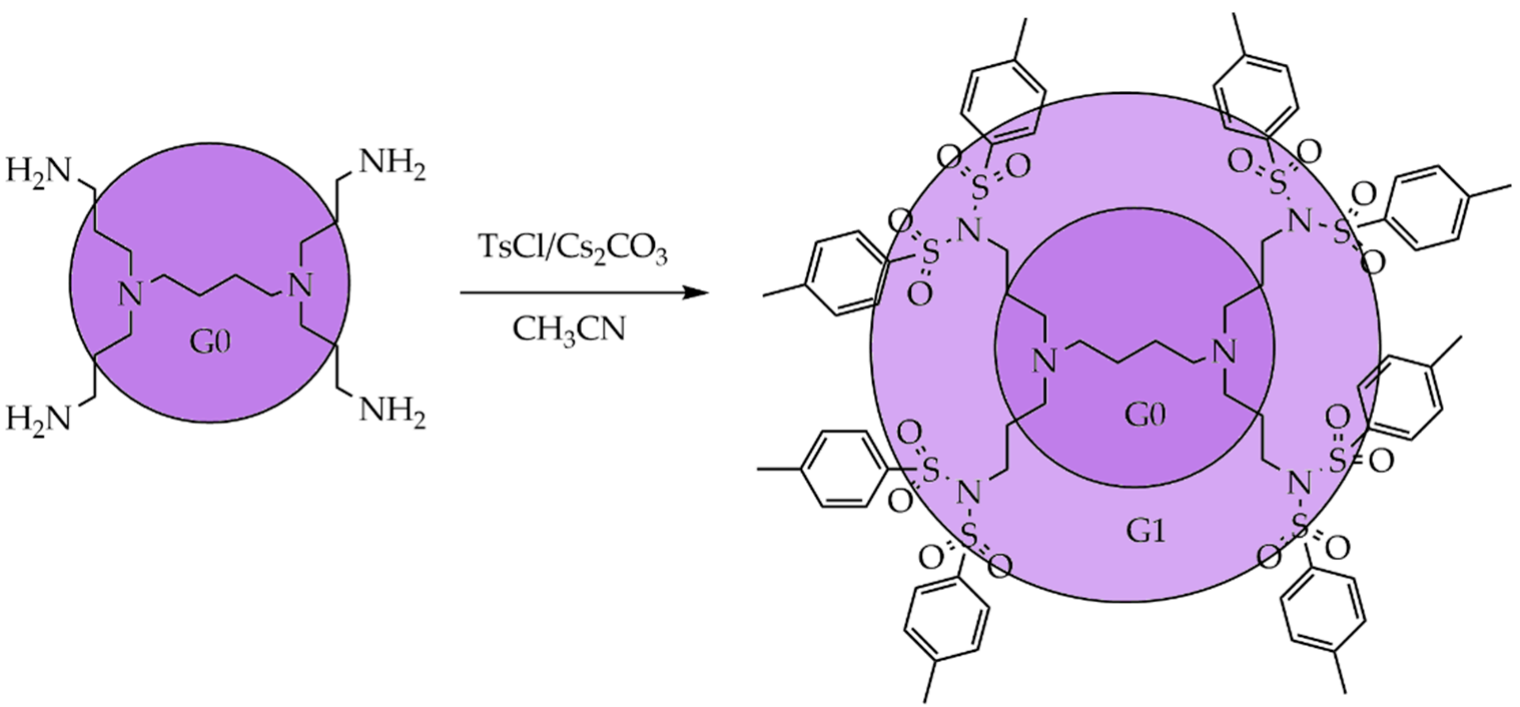
Persulfonylation of a G1 polypropylene-amine (POPAM) dendrimer.

The commercial availability and widespread popularity of polypropylene-amine dendrimers (POPAM) [43] and polyamidoamine dendrimers (PAMAM) [44] are the main factors for studying persulfonylation using these types of dendrimers to obtain higher-generation products. The interaction of the G1 POPAM dendrimers with tosyl chloride was carried out in the presence of cesium carbonate as a base to produce sulfonimide, as shown in Scheme 2 [45]. In addition to the target product, by-products of incomplete substitution of the hydrogen atom of the amino group can also form with the formation of a mixture of sulfonamides. Since the molecular weight of the unsubstituted products is compared to the target dendrimer, sulfonamides can be easily separated using conventional silica gel column chromatography.

**Scheme 3:**
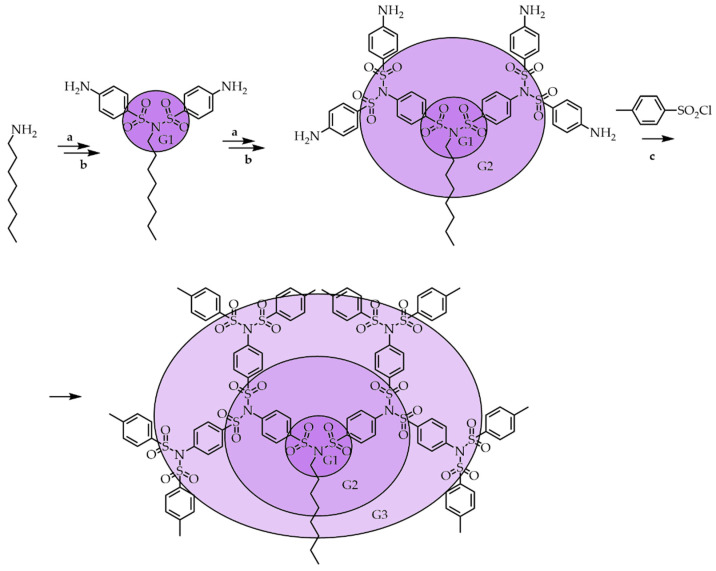
Synthesis of symmetrical oligo SBD (**a**) NO_2_C_6_H_4_SO_2_Cl, Et_3_N, CH_2_Cl_2_, reflux; (**b**) H_2_, 10% Pd/C, C_6_H_6_/C_2_H_5_OH (**c**) CH_3_C_6_H_4_SO_2_Cl, Et_3_N, CH_2_Cl_2_, reflux.

Interestingly, subjecting the G2 POPAM dendrimer to the same sulfonylation procedure with tosyl chloride does not lead to the expected products. Under these conditions, further sulfonylation of the G2 POPAM dendrimer yielded a complex mixture of products consisting of various sulfonylated fragments not subject to separation and purification. Similar to POPAM dendrimers, outer shell PAMAM dendrimers contain aliphatic amino groups. However, the sulfonylation of the G2 PAMAM dendrimers with sulfonyl chlorides in the presence of cesium carbonate resulted in octasulfonamide rather than hexadecasulfonimide. The method proved to be of limited use because it could only be applied for the peripheral decoration of G1 branched oligoamines. The partial persulfonylation of the oligoamines makes product separation difficult. Therefore, future investigations on the sulfonylation of amine-terminated dendrimers of other sulfonyl chlorides may lead to the development of new approaches to selective functionalization with the creation of new dendritic structures with useful properties. Based on the previous N-bis-sulfonylation (persulfonylation) results, Oleg Lukin et al. described the synthesis of a newly designed type of dendron that carries sulfonimide functional groups at every branching point. The selectivity of the primary amines persulfonylation in combination with a repeating methodology allows the creation of selectively decorated and shape-changing dendritic sulfonimide structures from simple building blocks [6]. Taking into account the limitations of the peripheral decoration of aliphatic amines, Oleg Lukin et al. employed an amine persulfonylation, which results in a 1→2 branching addition in a repeating manner that would lead to dendritic sulfonimides. The synthetic procedures are shown in Scheme 3 [6]. The main reactions involved are the persulfonylation of amines and the reduction of nitroaromatic compounds. 

Oligosulfonimides were synthesized in two steps: by treating N-octylamine with sulfonyl chloride with the nitro group in the presence of triethylamine and catalytic reduction of the nitro moieties to the corresponding amines. Octylamine was selected as the core molecule because of the good solubility of intermediate sulfonimides compared to similar primary amines with different alkyl chains [46]. The octylamine was persulfonylated in one step using an excess of the corresponding sulfonyl chloride. 

**Scheme 4:**
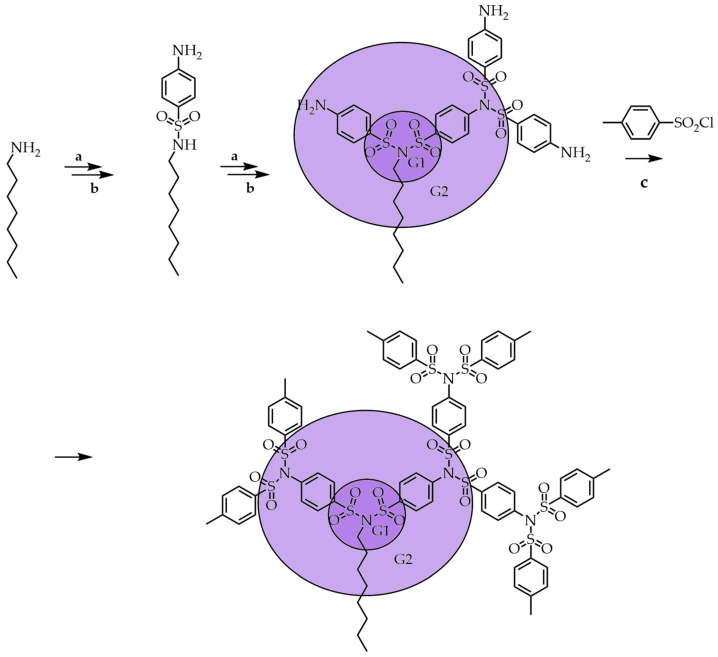
Synthesis of SBDs with structural asymmetry. (**a**) 4-NO_2_C_6_H_4_SO_2_Cl, Et_3_N, CH_2_Cl_2_ (**b**) SnCl_2_, HCl, EtOH, reflux; (**c**) CH_3_C_6_H_4_SO_2_Cl, Et_3_N, CH_2_Cl_2_, reflux.

In the last step, the dendrimer was functionalized by persulfonylation with chemically inert tosyl chloride, giving a G3 symmetrical dendrimer with methyl groups at the ends. Thus, the divergent approach shown in Scheme 3 is one of the most effective methods for producing repeating branched structures based on sulfonimide with various functional groups at the ends. Another advantage of this method is the simplicity of the procedure for purification of the obtained dendrimers by simple recrystallization in suitable solvents with a satisfactory yield. The synthesis of SBDs is attractive from the point of view of the commercial availability of various arylsulfonyl chlorides, which allows one to quickly obtain different types of high-generation SBDs decorated with the desired functional groups. Compared with previous works, this new one-step persulfonylation for primary amines by only heating with an excess of both the arylsulfonyl chlorides and bases affords much better yields than the reaction requiring a longer reaction time with cesium carbonate in acetonitrile. The ability to control the sulfonylation reaction allows one to obtain dendrimers having incompletely substituted branches in their structure; the presence of such defects can be programmed for certain tasks. In Scheme 4 [6], at the first stage, the only sulfonamide was prepared from octylamine and then further sulfonylated with a large excess of sulfonyl chloride, which led to full persulfonylation. The repetition of these steps afforded unsymmetrical sulfonimide.

Intermediate sulfonamide can be sulfonylated at the arylamine position or at the sulfonamide position with different sulfonyl chlorides to obtain an asymmetrically branched structure. The first molecule of sulfonyl chloride reacts with the amino group, and then the second molecule of sulfonyl chloride reacts in the same position, even if the sulfonamide position is present in the molecule. This selectivity can be explained based on fundamental organic chemistry; the amino group attached to the aromatic ring is more nucleophilic than the sulfonamide (–SO_2_NH) because its nitrogen lone pair is delocalized in the amide bond. Due to unsymmetrical synthesis with the incorporation of different functionalities, dendrimers with similar interiors but different exteriors called Janus-type dendrimers [47] were obtained. This synthetic approach has the advantage of having a wide range of affordable, inexpensive sulfonyl chloride reagents that can be used to produce chemically stable SBDs of various designs that do not require sophisticated purification methods. Despite this advantage, several shortcomings were limiting the synthesis of SBDs. For example, with an increase in dendrimer generation, its molecular weight also increases, which can significantly reduce the product’s solubility and prevent further synthesis and the purification procedure of the target product. In addition, the catalytic reduction reaction was not fully reproducible and led to an increase in the number of defects and by-products. The reduction of p-nosyldecorated dendritic sulfonimides with tin chloride (SnCl_2_) instead of Pd or Raney-Ni catalysts in boiling EtOH gives a full reduction but with subsequent cleavage of the p-aminobenzenesulfonyl groups to quantitatively form the corresponding amino sulfonamides. It was found that the use of a mixture of EtOH and CH_2_Cl_2_ in equal proportions is the way to the pure reduction of nitro groups. To reach a higher generation of SBDs, Oleg Lukin et al. investigated the influence of different arylsulfonyl chlorides under the same reaction conditions. Investigations indicated that para- and meta-substituted arylsulfonyl chlorides that lead to G3 and G4 compounds are usually high yielding. The reactivity of aromatic and aliphatic sulfonamides and aromatic amines is different and decreases with the increasing aliphatic part: ArSO_2_NHAr ‘> ArNH_2_> ArSO_2_NH-Alk [48].

In 2010, a group of chemists described the synthesis of G1 and G2 methacrylate monomers containing sulfonimide units at each branch and their radical polymerization to the corresponding sulfonimide-based polymers [36].

**Scheme 5:**
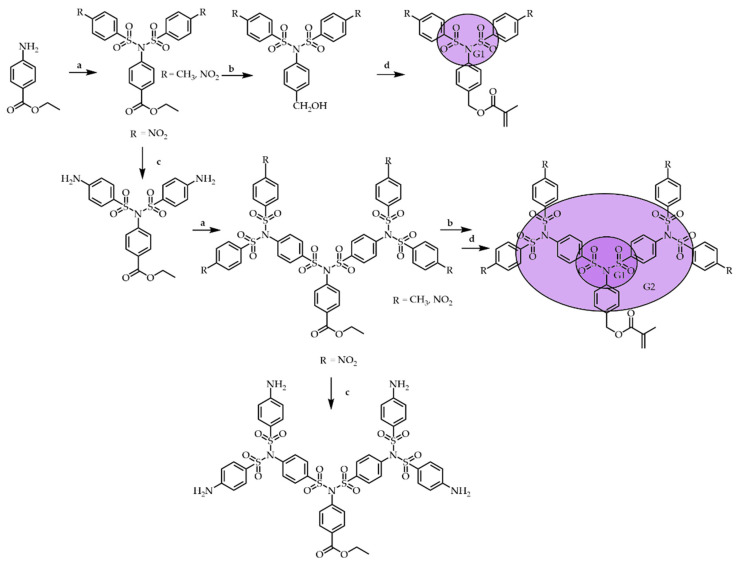
Synthesis of macromonomers MG1 and MG2. (**a**) ArSO_2_Cl, Et_3_N, CH_2_Cl_2_ (**b**) diisobutylaluminum hydride (DIBAL-H), tetrahydrofuran (THF) (**c**) SnCl_2_/HCl, EtOH: dichloromethane (DCM); (**d**) MAC, trimethylamine (TEA), THF.

Scheme 5 [36] highlights the main reactions, including the sulfonylation of amino groups and the reduction of nitro groups and esters. The G1 monomers’ syntheses started from commercially available ester, which was sulfonylated with arylsulfonyl chlorides. The ester groups were cleanly reduced with diisobutylaluminum hydride (DIBAL-H), affording corresponding alcohols. In the last step, methacrylate was added by reacting alcohols with methacrylic acid chloride (MAC). The synthesis procedures of monomers of the G2 and G3 were similar to the above. These procedures can be repeated, leading to the formation of a G3 monomer MG3(CH_3_). For the polymerization reaction, the monomers were dissolved in DMF, and the solution was heated under nitrogen in the presence of azoisobutyronitrile (AIBN). The highest G5 SBD decorated with 32 naphthyl terminal groups was prepared using divergent techniques [20] with two steps, similar to that mentioned in Scheme 3. 3-Methoxypropylamine was used as the starting material and persulfonylated with p-nosyl sulfonyl chloride. The nitroaromatic compounds were reduced with tin (II) chloride because the use of other reducing reagents, such as Fe/NH4Cl, Zn/CH_3_COOH, and others, led to mixtures of products. The two-stage approach has significantly increased the production of higher-generation SBDs. Thus, the G3 was obtained on a scale of 7 g in one synthetic cycle, while the species of the G4 were obtained on a scale of 5 g, and the G5 SBDs shown in Table 1 was obtained on a 2 g scale [20].

### 3.2. Convergent Technique

**Scheme 6:**
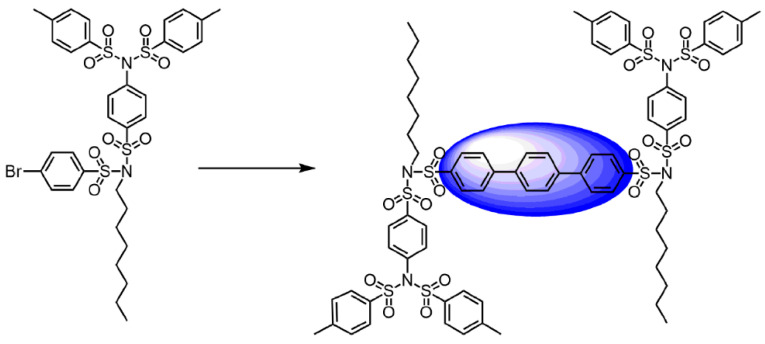
Synthesis of a terphenyl-cored SBDs using 1,4-phenylene diboronic acid diester, Pd[(P(pTol)3]3, THF/H_2_O.

Typically, SBDs, as well-known commercial available dendrimers such as PAMAM and POPAM, are synthesized using a divergent procedure [49], which has the disadvantage of significantly reducing the yield with increasing dendrimer generation and the accumulation of statistical defects in higher generations. A convergent procedure is usually used to simplify the synthesis and purification and provide better yields of high-generation dendrimers. A convergent approach to the preparation of dendrimer structures is based on the production of branched compounds bearing the active functional group, which can be involved in covalent attachment with the core molecule. A vast number of multifunctional compounds can be used as cores for dendrimers, such as biphenyl, terphenyl, pentaphenylene, and triazine core molecules. Scheme 6 [6] shows the use of tosyl-decorated sulfonimide dendrons in the synthesis of terphenyl-cored dendrimers using double Suzuki cross-coupling (SCC) reaction.

Surprisingly, sulfonimide derivatives with nitroaromatic groups on the periphery do not react under Suzuki conditions. Although there were several reports of dendrimers obtained by such a one-stage assembly method, they did not receive further development due to the following restrictions: getting hyperbranched polymers [50] with an increase in molecular weight (>2 kDa) instead of dendrimers, or poor chemical stability [51]. Thus, the development of simple methods for obtaining structurally advanced high molecular weight dendrimers remains a problem. Using the previously reported stepwise persulfonylation of octylamine with various sulfonyl chlorides by sequential reactions, a G4 dendron with a 4-bromobiphenylsulfonyl unit in the focal position was achieved. The double cross-coupling reaction of the dendrons with 1,4-phenylenediboronic acid can give the pentaphenylene cored dendrimer with a molar mass exceeding 10 kDa [52].

**Scheme 7:**
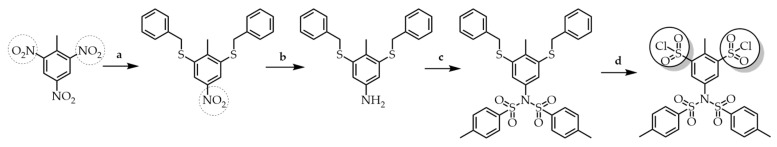
Synthesis of bis-sulfonyl chloride. (**a**) benzyl mercaptan, K_2_CO_3_, DMF (**b**) SnCl_2_, i-PrOH (**c**) CH_3_C_6_H_4_SO_2_Cl, Et_3_N, CH_2_Cl_2_ (**d**) N-chlorosuccinimide, HCl, CH_3_COOH.

Rozhkov et al. proposed the synthesis of new branched arylsulfonyl chlorides, which can be further used as reagents for the convergent synthesis of SBDs [53]. As shown in Scheme 7, the synthesis begins with the target molecule, in which the more reactive nitro groups located in the ortho position are used for substitution. The para position’s remaining nitro group was reduced to the amine group using tin (II) chloride. The amine was persulfonylated in a single step with tosyl chloride to give the sulfonimide, which was further converted to bis-sulfonyl chloride with a chlorinating reagent.

The new sulfonimide branched arylsulfonyl chlorides, which can be useful reagents for the convergent synthesis of multifunctionalized core compounds, were obtained in good yield and purity. [53] The convergent route reaches its limit at G3 species, whereas the divergent approach can be used to prepare higher generation sulfonimide-based dendrons. 

### 3.3. Click Chemistry

**Scheme 8:**
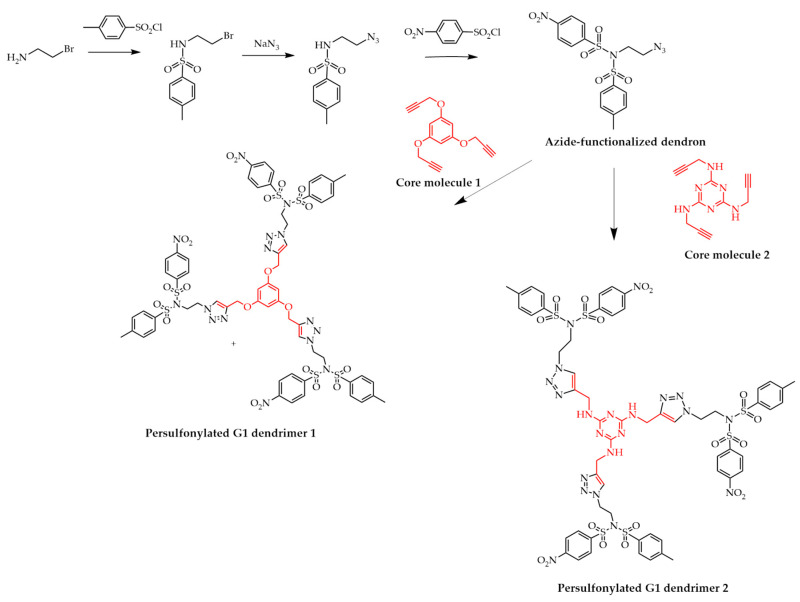
Synthesis of persulfonylated azide-functionalized dendron and the convergent synthesis of persulfonylated G1 dendrimers.

Tewari et al. synthesized azide-functionalized persulfonylated termini with sulfonimide groups, as outlined in Scheme 8 [54], which were linked together using click chemistry [55]. However, only the G1 dendrimers were obtained using this procedure, emphasizing the importance of the initial divergent or convergent approaches. 

The synthesis of dendrimers and dendrons with sulfonimide units at each branch point is based on a series of repeating sulfonylation reactions of amines and sulfonamides followed by reduction of the nitro group into an amino group. Such a combination allows precise control of the form of dendrimers, the number of branches in each generation, and their peripheral design with functional groups. Using the selectivity inherent in reactions eliminates the sophisticated protection strategy of the group. Currently, the construction and functionalization of dendrimers have been achieved through a set of reactions that are high yielding, fast, inexpensive, and operationally simple. Therefore, sulfone-based moieties in that category, especially N-monosulfonylation and N-bissulfonylation-based reactions, fulfill all the high-efficiency criteria and cost-effective novel dendrimer synthesis. Nevertheless, the synthesis of SBDs is limited to the G5 and requires further development. Since there has been exponential growth in newly synthesized chemical compounds [56], their preparation methods are still essential, and research and development aimed at improving the synthesis have not lost their relevance. The lack of chemical diversity of SBDs and the limited synthesis methods currently available show that the chemistry of SBDs has not been sufficiently studied. In addition to increasing the diversity of structures and increasing the yield of synthesis reactions, special attention must be paid to dendrimers with large aromatic and form-stable branches. It is expected that such rigid, inert structures will have high melting and glass transition temperatures, opening up new possibilities for chemical modification and application. Despite numerous works on the synthesis of dendrimers, much work remains to be done in this regard. As indicated by the number of works and the results obtained, the divergent method remains the preferred synthesis method today, but this does not reduce the importance of other approaches. There is still a need for even higher generations of all types of dendrimers, as well as for high-performance and optimized syntheses of individual families of dendrimers to access targeted mono- and multifunctionalized products. The development of various new and innovative building blocks for the synthesis of dendrimers is also important.

## 4. Purification and Separation

SBDs are easy to handle and do not require difficult purification procedures. By removing the molecules with defects in their structures, the purification process improves the material’s monodispersity. This is important because the presence of defect structures due to incomplete synthetic reactions or by-processes during the divergent synthesis can affect the observed material properties. Notably, chromatographic purification can be used for SBDs starting with the G3 species; all dendrimeric molecules of lower generations require recrystallization [48]. As already mentioned, these dendrimers are mostly obtained by divergent techniques, which can lead to defects in high-generation dendrimers occurring from incomplete addition reactions during the synthesis or from side processes. Therefore, at some stages, a mixture of products with different molecular weights can be obtained. Some techniques can be used for the separation of a mixture of dendrimers, such as gel permeation chromatography (GPC), high-performance liquid chromatography (HPLC), and simple thin-layer chromatography (TLC) [57]. GPC is impractical for the separation of mixtures of isographic and non-isographic isomers. Unlike GPC, HPLC with a preparative column can be used to separate isographic isomers but with very low efficiency, and it is not applicable for non-isographic isomers. The same situation is seen for silica gel thin-layer chromatography (TLC). Non-isographic isomers have the equal R_f_ value, and separation is impossible [7]. In contrast, a mixture of the isographic isomers showed noticeable separation on a TLC plate. This implies that the isographic isomers have not only different hydrodynamic volumes but also different polarities and affinities for the stationary phase.

## 5. Activation of SBDs

**Scheme 9:**
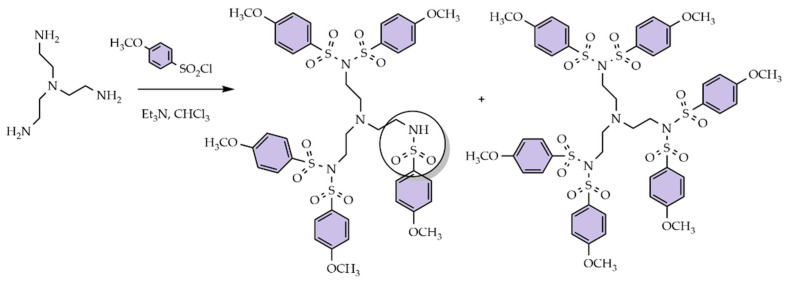
Synthesis of mixed sulfonimide and sulfonamide derivatives.

For SBDs, it is possible to create derivatives containing partially unsubstituted branches. Thus, in addition to sulfonimide groups, sulfonamide groups are formed. This leads to the dendrimer’s activation and allows its use as a derivative for the synthesis of more diverse structures with great functionality. One way to create such mixed structures is through slow persulfonylation, leading to incomplete substitutions. In most cases, persulfonylation with a large excess of reagents proceeds quickly, which leads to persulfonylated products. However, an exciting exception was found for the reaction of tris(2-aminoethyl) amine with p-methoxybenzenesulfonyl chloride, which is depicted in Scheme 9 [41]. In this case, the introduction of the sixth sulfonyl unit occurs very slowly, and we can see a mixture of equal amounts of persulfonylated and incompletely persulfonylated products. 

**Scheme 10:**
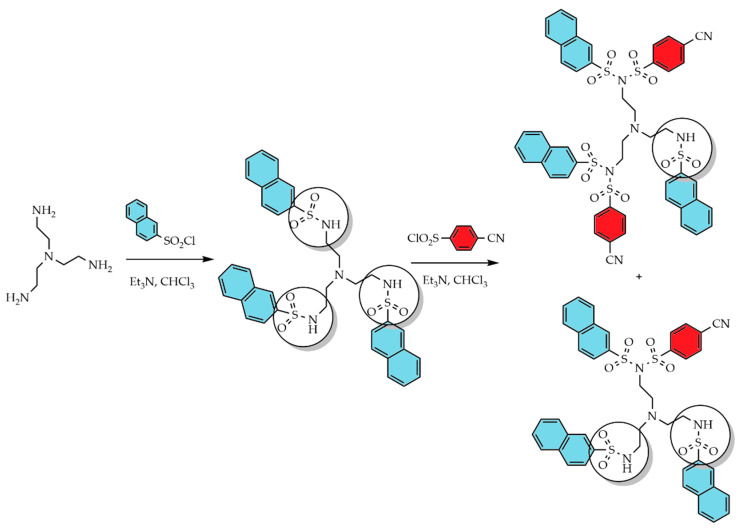
The synthesis of mixed sulfonimide and sulfonamide derivatives with different functionalities.

Even more interesting results were obtained in the persulfonylation of threesulfonylated product with p-cyanobenzenesulfonyl chloride, which occurred smoothly at room temperature, and only mono- and bis-sulfonimides were isolated. In contrast, complete persulfonylation of the threesulfonylated product could be achieved in boiling acetonitrile. The synthesis is shown in Scheme 10 [41].

**Scheme 11:**
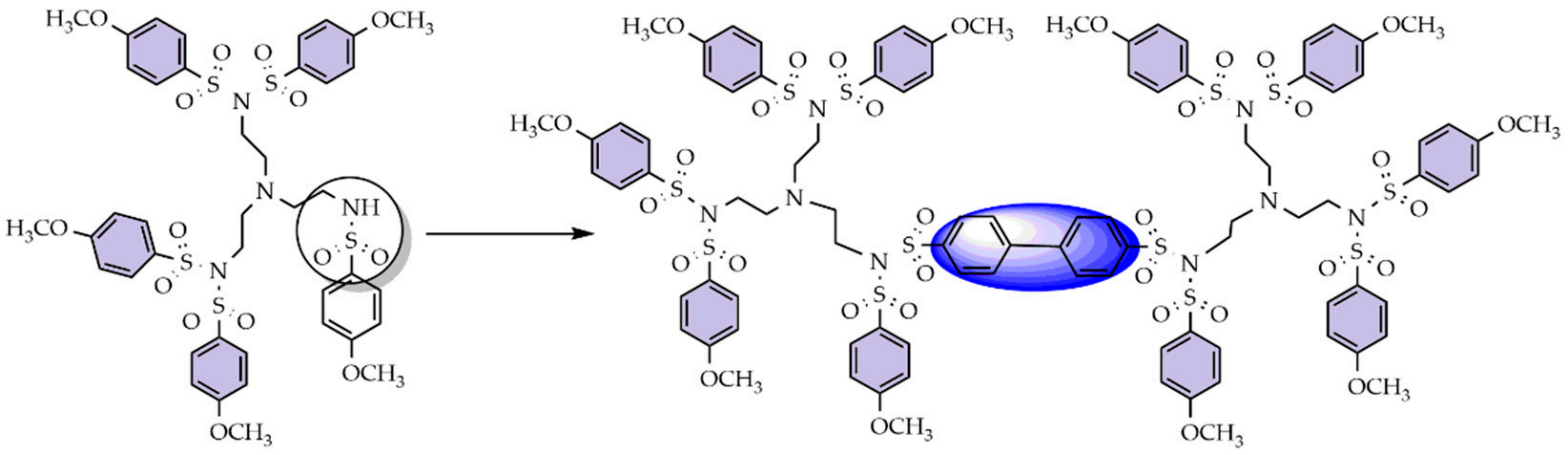
The synthesis of a dumbbell-shaped dendrimer by a convergent method.

The coupling of monosulfonamides with 4,4′-biphenyldisulfonyl chloride led to a dumbbell-shaped dendrimer, as outlined in Scheme 11 [41].

**Scheme 12:**
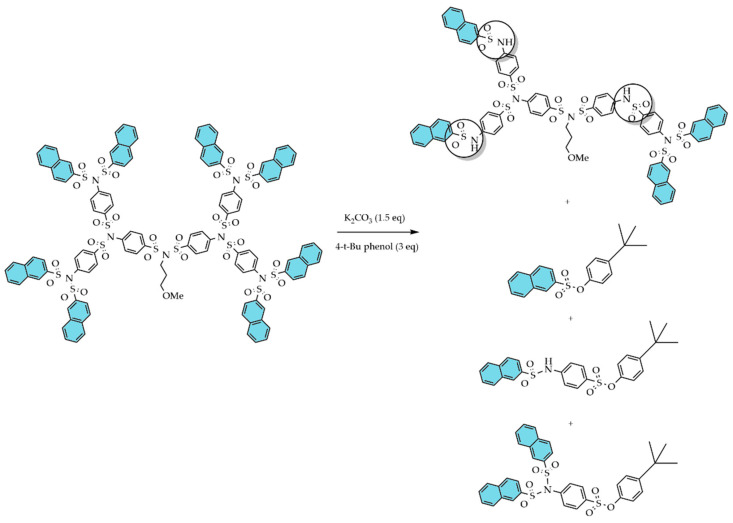
Partial removal of the branches from naphthyl-decorated dendron by the action of a phenolate nucleophile.

Another way to create structures bearing sulfonamide groups is based on the partial degradation of existing SBDs. SBDs can lose some of their branches when heated in the presence of nucleophiles such as thiolates and phenolates. As exemplified in Scheme 12, a reaction of naphthyl-decorated dendrimer with tert-butylphenol in the presence of potassium carbonate resulted in a number of products, including a compound bearing three sulfonamide functionalities, as the major product [58]. In addition to the major compound, three products from the nucleophilic displacement of the sulfonimide sulfur atom were isolated and characterized. Interestingly, nucleophilic attacks occurred only at aromatic sulfonimide branching points, while the aliphatic sulfonimide of the core remained intact.

**Scheme 13:**
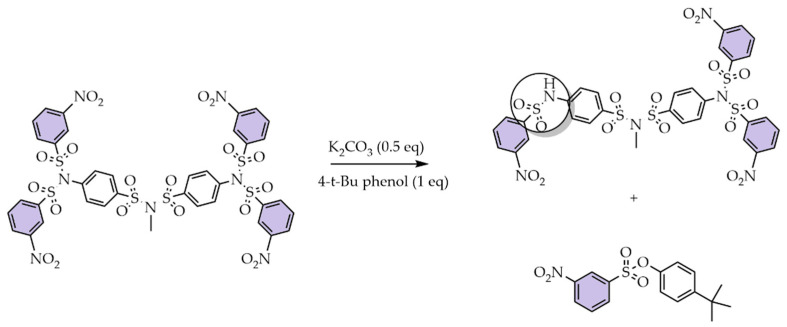
Selective removal of one peripheral group from G2 dendron.

**Scheme 14:**
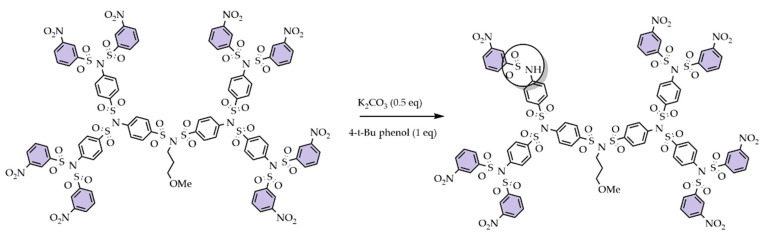
Selective removal of one peripheral group from G3 dendron.

The partial cleavage of a perfectly symmetrical dendrimer is known as dendrimer activation and is used to produce hyperbranched polymers for gene transfection and ink jet-printing applications [59]. The activated dendrimers contain several sulfonamide fragments, which are considerably more hydrophilic than the parent sulfonimides. To check whether the cleavage of individual branches can proceed in a more controlled fashion, the reactions of G2 and G3 dendrimers were carried out. As shown in Scheme 13, the reaction of an m-nitrobenzene-decorated dendrimer with one equivalent of p-tert-butylphenol in the presence of 0.5 equivalents of potassium carbonate resulted in the clean removal of only one peripheral group, giving monosulfonamide and sulfonate, which is generated by nucleophilic displacement of the sulfonimide sulfur atom. The same reaction of G3 dendrimer (Scheme 14) yielded monosulfonamide and sulfonate [6].

Individual branches can be cleaved in a controlled fashion for G2 and G3 dendrimers. 

## 6. Characterization

One of the most common and widely used techniques for dendrimer characterization is proton and carbon nuclear magnetic resonance (NMR) spectroscopy [60]. ^1^H and ^13^C NMR methods are commonly used to study the structural details of organic compounds; depending on the content of certain chemical elements, one can use techniques such as ^15^N NMR and ^31^P NMR spectroscopy. NMR spectroscopy is useful for the characterization of intermediates in multistep syntheses to monitor the completeness of the reaction and quality of the product. Although many signals of the dendrimers have very similar chemical shifts in the aromatic region of the ^1^H NMR spectra, in the case of naphthyl decorated dendrimers, the signals for the naphthyl α-protons and ß-protons are highly indicative. In many cases, correlation NMR spectroscopy must be used to confirm a particular structure (e.g., nuclear Overhauser effect spectroscopy, NOESY). In general, NMR analysis can help identify the presence of structural defects. Still, the exact nature of defects can be challenging to determine because peaks are often broad due to a very similar environment. 

Another important method of analysis is mass spectrometry, which provides not only information about the precise molecular weight but also the possible presence of defects in the structure. Mass spectrometry is a common tool for the analytical characterization of dendrimers. The presence of signals corresponding to lower molecular weights may indicate incomplete reactions during synthesis and the absence of some branches of the dendrimer. Matrix-assisted laser desorption/ionization (MALDI) has been greatly developed [61]. This technique involves desorption “soft” ionization caused by laser radiation’s action on the matrix with the analyzed sample. The matrix is selected in a way to minimize the destructive effect of the laser on the substance. On the other hand, the matrix material enhances ionization, which, in turn, leads to an increase in the accuracy of the results. In addition, electrospray ionization (ESI) is often used to transfer the dendrimer to the gas phase and subsequent particle analysis [62]. Possible defects in the structure of the dendrimer can be detected using mass spectrometry analysis with the correct consideration of possible ionization defects. The reaction of dendrimers with acidic matrices, for example, 2,5-dihydroxy benzoic acid [63], can occur during the MALDI process, which can lead to the cleavage of sulfonimide groups and the generation of sulfonamides. Thus, defects arising from ionization can be identical to those arising from incomplete substitution. To eliminate errors, it is necessary to study samples using two methods using different matrices. Mass spectrometry can give not only a quantitative characteristic but also a qualitative one, for example, protonation sites [64] or weak and noncovalent complexes with a dendrimer molecule [65].

Understanding the spatial arrangement of the branches of dendrimers is of great importance because their optimal geometry determines the shape and functionality of the molecule, solubility, and the behavior of the molecule at the interface. Molecular modeling techniques may provide some insight into the location of the molecule in space and the influence of the medium. Still, they are limited by the size of the units and the available conformational space. Another method for describing the structure is X-ray crystallography, but not all dendrimer molecules can form crystals, although some work in this direction has been carried out. The dendritic structures have a tendency to form crystal structures; for example, there were only a few single-crystal X-ray structures of G1 [66] and G2 Frechet-type dendrons [67], and no crystal structures of higher-generation dendrons are available in the literature. The available literature on crystallographic studies related to sulfonimides include metal complexes of trifluoromethylsulfonimide ligands [68] and alkylsulfonimide bridges incorporated into [2.2]-paracyclophane structures [69]. Concerning SBDs, crystals of some dendrimers were obtained, for example, a dendrimer decorated with nitro groups can be recrystallized from CHCl_3_ or THF to obtain amorphous colorless powders. In contrast, dendrimers decorated with tosyl and 2-naphthyl mixtures can be precipitated CH_2_Cl_2_/MeOH in the form of tiny colorless mixtures in the form of needle crystals. In 2009, a group of scientists showed that SBDs could form crystals and identified and described a set of 13 single-crystal structures of G1 and G2 SBDs [36]. SBDs are capable of regular reproducible packaging in a crystalline state. The infinite structural diversity of SBDs makes them very promising materials for the study of crystal formation [70]. Single-crystal X-ray analyses of several SBDs with naphthyl terminal groups have been performed [71]. The parallel π-stacking intermolecular interaction of naphthyl groups is often observed in crystal structures of different aromatic compounds [72] and crystal structures with a combination of aromatic and perfluoroaromatic rings [73] and other combinations of electron-rich and deficient aromatic compounds [74]. The absorption spectra of SBDs in CH_2_Cl_2_ solution show an intense band with a maximum at ≈240 nm because of the absorption by the sulfonimide groups. With an increase in the number of arylsulfonyl units, the intensity of this band increases linearly [52].

The structure of the dendrimer was confirmed by elemental analysis and IR spectroscopy. The bands at 1487 and 1605 cm^−1^ are due to the stretching vibrations of aromatic C=C bonds, and the bands at 673, 713, 755, and 829 cm^−1^ are due to the bending vibrations of aromatic C–H bonds. Additionally, the absorption bands at 1360‒1330 and 1180‒1140 cm^−1^ are typical of the SO_2_ group in sulfonimides [75].

## 7. Application

In chemistry, the discovery of new materials with specified properties and the optimization of the conditions for their production and application are essential tasks of theoretical and practical interest [76]. Prospective materials are needed to enable future technological developments in different areas, such as sustainable energy-efficient processes, targeted drug delivery, sensor technology, and construction. The results of such development can be less expensive, lighter, more durable, or more functional materials, which can have a significant impact on our lives in the near future [77].

Dendrimers as new materials have made essential contributions in different fields, such as biomedicine, chemistry, material science, engineering, electronics, and optics [78]. It is challenging to distinguish between pre-existing practices and outstanding future applications of dendrimer chemistry. Dendrimers as independent monodisperse, symmetrically branched molecules [79], and also active branched dendrons [80,81] together with polymers based on their structure [82] can be attractive in many areas of fundamental, applied science [60], and industry. The regular structure of dendrimers and the ability to control their physicochemical properties have attracted significant interest in their further development, study, and practical application. The choice of peripheral groups allows the chemical, physical, and biological functionality of these macromolecules to be tuned. The outer shell containing functional groups form the periphery of dendrimers, the high branching, and the opportunity to design molecular structure make these materials of great interest in many sensing [83] and surface-active [84] applications. Moreover, dendrimers have nanoscale dimensions, globular surfaces, and powerful properties, such as low viscosity compared to polymer species, tunable solubility, and reactivity, in combination with unique functionalities, making them of interest in various fields. 

Existing applications of SBDs and potential applications in different planning stages are described below with respect to dendrimer properties. 

### 7.1. SBDs in Electrochemistry

Proton-conductive polymers are attractive materials as an alternative electrolyte for power generation fuel cells [85]. Membrane materials used in fuel cells must meet a number of requirements, for example, chemical and mechanical stability, low permeability, and good ionic conductivity, which significantly reduces the number of suitable materials. Previous studies have focused mainly on polymers containing sulfonic acid or perfluorosulfonic acid specific for Nafion [86]. Materials, containing sulfonimide groups, can be potential alternatives to materials based on sulfonic acid. Allcock et al. described the synthesis of a phenolic compound containing sulfonimide groups to prepare further a polymer containing a phosphorus–nitrogen double bond. The sulfonimide side group is non-nucleophilic, thus permitting its use as a replacement for classic macromolecular chlorine, which allows the final polymer properties to be controlled through the choice of substituents. The use of a sulfonimide polymer improves the mechanical properties, moderates water swelling, and enhances proton conductivity [87]. Sulfonimide groups have excellent thermal stability up to 370 °C and chemical stability. The additional fluorine presence in the sulfonimide polymer ensured the high stability of the polymer chain from oxidizing radicals. Therefore, further studies in the field of synthesis of sulfonimide-based membranes, which could serve as an alternative to Nafion^®^, are preferred today [88].

Another promising area requiring new electrolyte solutions is lithium-ion batteries, which are widely used for energy storage in portable electronics due to their low weight and energy density [89]. In this storage system, the electrolyte plays an essential role in battery performance. Organic liquid electrolytes currently in use can be explosive due to possible overheating [90]. Therefore, polymer electrolytes can be attractive alternatives due to their nonvolatility, proper safety, and thermal stability during cycling [91]. Gel single-ion polymer electrolytes (SPEs) based on polymers containing lithium imide salts [92], for example, bis (benzenesulfonyl) imide, attract considerable attention due to their good ionic conductivity and compatibility with electrodes. The strongly electron-withdrawing O=S=O group amplifies the electron delocalization on the nitrogen atom in synergy with the benzene ring in the para-position [93], which makes sulfonimides obtained by the simple radical copolymerization of 4-styrenesulfonyl (phenylsulfonyl) lithium imide (SSPSILi) with maleic anhydride (MA) attractive from the point of view of conductive materials. This alternating structure increases the speed and uniformity of the dissociation of lithium ions, improving ionic conductivity and a high amount of lithium-ion transfer at room temperature [94].

### 7.2. SBDs in Optics and Sensors

The opportunity to incorporate various photoactive organic units in structure of an SBD provides a new platform for applying such molecules in light-harvesting devices [95], sensing technologies with signal amplification [96], and fluorescence at the single-molecule level [97]. One such luminescent unit is terphenyl, which exhibits a fluorescence band in the shorter wavelength UV region with a high quantum yield [98]. The SBD with a terphenyl core was synthesized previously [6], and it showed higher steady-state anisotropy compared to starting terphenyl species [99]. In addition to the terphenyl nucleus, the search for other photoactive particles capable of increasing the quantum yield of fluorescence is relevant [78]. Moreover, the sulfonimide branches play an important role in slowing molecular rotation, affecting fluorescence depolarization. 

### 7.3. SBDs in Medicine

**Figure 5:**
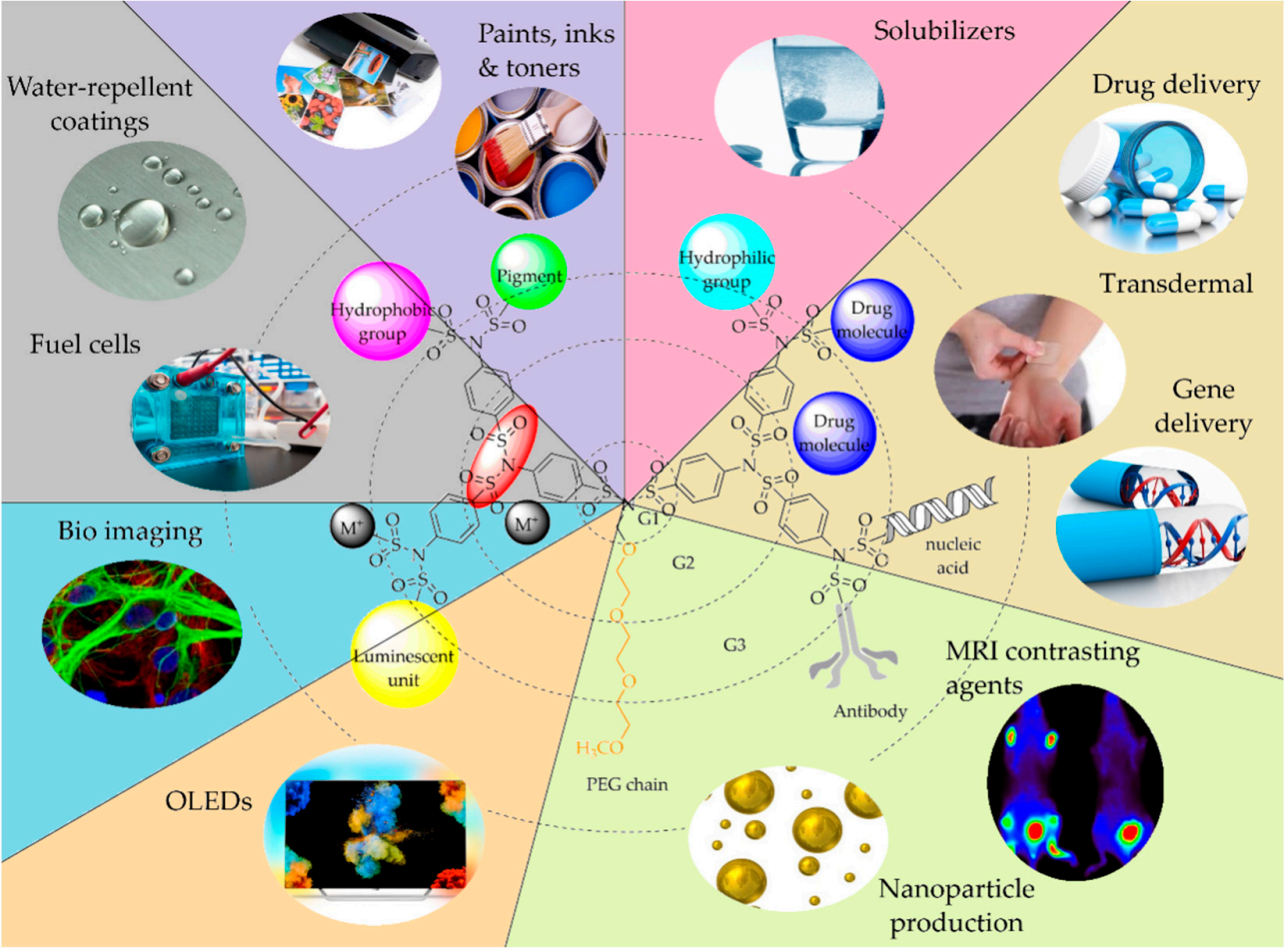
Application of SBDs according to the nature of functional groups and branching points.

Unlike polymers, structural accuracy and, therefore, more uniform SBD properties make these materials applicable in the biomedical industry. The targeted guest molecules can be attached to the periphery of the SBD via functional groups or located in the SBD’s internal voids. SBDs, which are the object of our study, have unique properties such as high glass transition (>130 °C) and melting temperatures (>150 °C) [36]. As mentioned above, the high melting and glass transition temperatures are preferable for future applications in medicine and pharmaceuticals for preparing consumable materials bactericidal properties, e.g., antiseptic napkins. Additionally, due to the multivalency of the SBD, it effectively adheres to different surfaces. As shown in Figure 5, the G3 and G4 SBDs can have several internal cavities, making it possible to accommodate guest molecules. 

### 7.4. SBDs as Additives, Printing Inks, and Paints

SBDs with monodisperse “tree-like” structures can be used as additives in toners for color printing [100]. Dendrimer additives regulate viscosity and increase adhesive properties [101], which allows effectively covering the surface of the paper, which is important for quality and inexpensive printing. Moreover, the use of such doped toners will allow the rational use of inks, which will significantly reduce paint consumption [102]. Companies producing printing inks are interested in dendrimer additives. Highly uniform ink adhesion to the surface can be achieved by adding a dendrimer, which will significantly reduce ink consumption and therefore reduce the cost of printing [103]. Moreover, in addition to sufficient adhesion to the surface, a dendrimer’s ability to form strong bonds with pigment molecules improves the print quality. Branched dendritic polymers are used to change the rheological properties of materials; for example, SBDs as additives may affect some materials’ viscosity and surface tension. Unlike linear polymers, the high molecular density of dendrimers makes them applicable as additives in dental chemistry. In dentistry, polymeric materials are required during photopolymerization, forming crosslinked structures filling the entire tooth hole’s entire volume, leaving no gaps between the fillings and the tooth. 

### 7.5. SBDs as Coatings

Another promising area for the use of sulfonimide-based crosslinked systems is the production of thin layers. An important feature of monolayers based on SBDs is their calibrated thickness, which is determined by the size of the original dendrimers. The high functionality and dense globular structure of SBDs suggest their use in materials such as coatings that possess unique barrier properties, chemically modified surfaces, and chemical sensors. In addition, the study of SBD behavior on surfaces provides new data on their physical and chemical properties and the features of their interactions with various types of surfaces. In addition, SBDs can be applied not only as monomeric building blocks for film formation but also because the functionalization of SBD with terminal groups can give the surface new properties. For example, the decoration of SBD with naphthyl groups can enhance the hydrophobicity of the molecules and, as a result, afford a surface with water-repellent properties. The pioneering work related to the formation of Langmuir–Blodgett thin films made up of G2, G3, G4, and G5 SBDs has been demonstrated recently [18]. The Langmuir–Blodgett method allows controlling dendrimer film formation and the required concentration, which is essential for real applications and gives films that are a few nanometers thick. Optical and electron microscopy methods and ellipsometry were used to study the morphology and structure of the obtained SBD films. The microstructures of all the samples showed similar cylindrical objects. Confirmation of the mechanical stability of thin films of SBDs was obtained by successfully transferring them to a copper mesh holder. The mechanical properties were evaluated by measuring Young’s modulus, which is a technique commonly used for the analysis of polymers. The obtained values of Young’s modulus for SBDs film referred to soft polymer materials. Contact angle measurements evaluated the wettability of the SBD films. The hydrophobicity of the films made from G2 to G5 materials depends on the number of hydrophobic groups and increased by 23%. The contact angle of the transferred films increased by 33° for the G5 SBDs and reached a maximum of 90°. In addition, the contact angles were dependent on the surface roughness.

## 8. Conclusions

Dendrimers’ diverse applications include gene therapy, chemical sensors, drug delivery systems, adhesive additives and coatings, light-harvesting materials, catalysts, electronic materials, and separating agents. The applications of dendrimers as additives in the field of catalysis, sensory technologies, and medical diagnostics are the most relevant and attract the most attention from researchers.

In this review, we fully described SBD synthesis and outlined their possible applications from biological to material science. We have described the types of dendrimers, their properties, characterization techniques, their importance, and the need for new SBDs and their derivatives. To date, several kinds of SBDs have been obtained using different reactions, but N-bis-sulfonylation (persulfonylation) is fast and clean. At present, the divergent synthesis of SBDs remains the most convenient and preferred, making it possible to obtain high generation dendrimers. By utilizing a two-step persulfonylation, it was possible to increase the yield of dendrimers and simplify the purification method compared to previously available methods. Both the synthetic strategies and application aspects can be enhanced by introducing heterocyclic, polycyclic hydrocarbons, heteroatoms, and various electron-rich or electron-deficient species along with the sulfonimides. Combined with advancing functionalities, these dendrimers will significantly impact the synthesis and applications of related species. Several dendritic assemblies possessing sulfonamide units together with sulfonimide functionalities have been synthesized. Such mixed sulfonimide/sulfonamide structures significantly expand both the synthetic possibilities and the scope of their applications.

We collected and organized all the information available on SBDs. We expect that the given information would not only acquaint researchers with the recent advances in the synthesis and applications of these dendrimers but also inspire them to use SBDs for the design of new molecules with enriched medicinal and material properties, leading to novel materials and composites for the benefit of humanity and technology.

## Figures and Tables

**Table 1:** Characteristics of naphthyl decorated SBDs.

Generations	Molecular Weight, Da	Dimensions, nm	2D Graphical Representation	3D Chemical Structure View ^1^
G2	1334	2.6	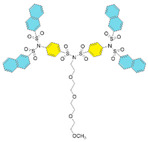	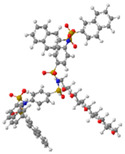
G3	2541	3.2	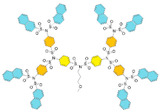	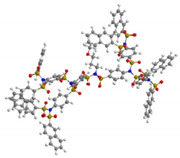
G4	5304	3.6	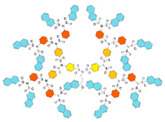	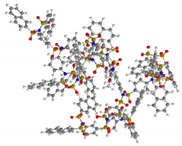
G5	10831	4.6	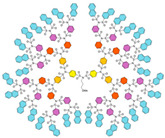	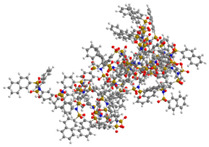

^1^ red—oxygen, yellow—sulfur, blue—nitrogen, grey—carbon, white—hydrogen.
